# Enablers and barriers of people with chronic musculoskeletal pain for engaging in telehealth interventions: protocol for a qualitative systematic review and meta-synthesis

**DOI:** 10.1186/s13643-020-01390-x

**Published:** 2020-05-31

**Authors:** Lívia G. Fernandes, Hemakumar Devan, Steven J. Kamper, Christopher M. Williams, Bruno T. Saragiotto

**Affiliations:** 1 Masters and Doctoral Programs in Physical Therapy, Universidade Cidade São Paulo (UNICID), São Paulo, Brazil; 2 Centre for Pain, Health, and Lifestyle (CPHL), New Lambton Heights, Australia; 3 Centre for Health, Activity and Rehabilitation Research (CHARR), School of Physiotherapy, University of Otago, Wellington, New Zealand; 4 Sydney School of Public Health, Faculty of Medicine and Health, The University of Sydney, Sydney, Australia; 5 Australia Institute for Musculoskeletal Health, Sydney, Australia; 6 School of Medicine and Public Health, Hunter Medical Research Institute, University of Newcastle, Newcastle, Australia; 7 Hunter New England Population Health, Hunter New England Local Health District, Wallsend, Australia

**Keywords:** Telehealth, Internet, Chronic pain, Musculoskeletal, Protocol

## Abstract

**Background:**

Chronic musculoskeletal pain represents an enormous burden in society. Best-practice care for chronic musculoskeletal pain suggests adoption of self-management strategies. Telehealth interventions (e.g., videoconferencing) are a promising approach to promote self-management and have the potential to overcome geographical barriers between patient and care providers. Understanding patient perspectives will inform and identify practical challenges towards applying the self-management strategies delivered via telehealth to everyday lives. The aim of this study is to synthesize the perceptions of individuals with musculoskeletal pain with regards to enablers and barriers to engaging in telehealth interventions for chronic musculoskeletal pain self-management.

**Methods:**

A systematic review of qualitative studies will be performed based on searches of MEDLINE, EMBASE, CINAHL, LILACS, and PsycINFO databases. Screening of identified titles will be conducted by two independent investigators. Data extraction will retrieve detailed qualitative information from selected articles. The critical appraisal skills program (CASP) checklist will be used for critical appraisal of included studies, and the level of confidence in the findings will be assessed using the confidence in the evidence from reviews of qualitative research (GRADE-CERQual). A thematic synthesis approach will be used to derive analytical themes.

**Discussion:**

This review will systematically identify, synthesize, and present enablers and barriers reported by people with musculoskeletal pain to engage in telehealth interventions. The review will provide information required to support the design and improvement of telehealth services.

**Systematic review registration:**

PROSPERO CRD42019136148

## Background

The International Association of Study of Pain (IASP) defines pain as an unpleasant experience that may or may not be linked to actual tissue damage [1]. Chronic pain is defined as pain lasting longer than the expected time for tissue recovery (around 3 months) [2]. Chronic musculoskeletal pain, such as osteoarthritis, back and neck pain, are the leading causes of years lived with disability across the world according to the Global Burden of Disease Study (2016) [3, 4]. In both high- and low-income countries these conditions have shown increased prevalence over the past two decades, illustrating the challenges of their management [4, 5]. Barriers reported by clinicians (e.g., consultation time, resources) and patients (e.g., service availability, geographical location) in translating evidence-based recommendations into practice contribute to the growing burden of musculoskeletal pain [6–8].

Best practice recommends self-management strategies (including education and exercise) for optimizing the management of musculoskeletal pain [8, 9]. Engaging with this type of treatment demands an active participation from the patient towards a lifestyle change, knowledge, and involves shared decision-making processes with clinicians [8, 10]. Telehealth is a promising mode of delivery for self-management strategies [7, 11]. Telehealth includes the use of technologies and related services (e.g., telephone, virtual reality, videoconference, apps, websites) to allow interactions (synchronous/real-time, e.g., videoconference, and/or asynchronous/store-forward, e.g., digital images) between healthcare providers and patients [12]. Telehealth interventions bring important value to health care programs as they overcome geographical barriers between patients and clinicians [13].

The evidence for telehealth interventions in improving musculoskeletal pain-related outcomes is favorable, and the results are comparable to face-to-face interventions [7, 14]. A recent systematic review of 13 randomized controlled trials found that telehealth interventions are as effective as usual/face-to-face care interventions for improving pain and function in people with musculoskeletal conditions [15]. O’Brien et al. [7] reported a small positive effect on pain and disability after reviewing the effectiveness of telehealth interventions compared to usual/face-to-face care for musculoskeletal pain. Healthcare providers believe online resources as a useful adjunct to face-to-face delivered treatments for chronic pain [16, 17]. Patients also have favorable attitudes to telehealth approaches of healthcare delivery. Feeling of “closeness at a distance,” independence, and improved knowledge about their “body and self” was perceived by patients who participated in a telehealth program after shoulder joint replacement [18]. Previous studies also found good patient satisfaction rates in telehealth interventions based on cognitive behavioral therapy and exercise and pain-coping intervention [19, 20].

Despite the promise of telehealth interventions, the implementation of technology within health systems as an alternative means for delivering care remains a challenge [6]. Patient and public engagement are a major issue for the expansion of telehealth [21]. Difficulties guaranteeing good internet access to remote areas, or access to adequate devices [22], as well as lack of acceptance from the elderly population [18], and poor interaction with web-based sources of information [23], have been identified as barriers to uptake of telehealth interventions. Low educational level and low income were also associated with inadequate internet access and use [24].

To maximize the access to, uptake and use of telehealth interventions in musculoskeletal pain management, it is important to understand patient perspectives towards engaging with these types of healthcare delivery. Understanding patient perspectives will inform and identify practical challenges towards implementing telehealth strategies in the future. There has been no comprehensive review of available evidence regarding patients’ perception towards telehealth. This systematic review aims to synthesize the perceptions of individuals with musculoskeletal pain with regards to enablers and barriers to engaging in telehealth interventions for chronic pain self-management.

## Methods

The review protocol was registered at the PROSPERO database of systematic reviews. The protocol was developed in accordance with the Preferred Reporting Items for Systematic Reviews and Meta-analysis Protocols (PRISMA-P) checklist [25].

### Search strategy

Electronic databases will include MEDLINE, EMBASE, CINAHL, LILACS, and PsycINFO. Forward and backward citation searches of included articles and relevant systematic reviews will also be conducted. There will be no language and search period restriction. Detailed search strategy is presented in Appendix.

### Eligibility criteria

Individuals presenting with any previously diagnosed or self-reported chronic musculoskeletal pain condition (e.g., back pain, neck pain, osteoarthritis), including post-surgical procedures due to a primary musculoskeletal condition. The definition of chronic pain will be aligned with ICD-11 classification [2] and will focus on conditions related to the musculoskeletal system, including diagnoses that can be subsumed under a different category (e.g., chronic widespread pain), aiming to include a wide range of studies and conditions. Inclusion criteria following the SPIDER format is available in Table 1 [26].

Studies that seek to guide telehealth intervention development by exploring patients’ opinions regarding what interventions should involve will be excluded. Mixed methods and qualitative studies primarily using quantitative data analysis approaches will be excluded.

**Table 1:** The SPIDER criteria adopted for selection of included studies

Sample	Individuals with chronic musculoskeletal pain (more than 3 months)
Phenomenon of interest	Telehealth interventions, using telecommunication networks as a means to deliver care (e.g., exercises, education, self-management strategies, counseling, cognitive behavioral therapy)
Design	Qualitative data both verbatim and edited by researchers, with or without the addition of questionnaires
Evaluation	Patients’ perceptions (enablers and barriers) on engaging with telehealth interventions, qualitatively described
Research type	Qualitative studies (qualitative data collection method and qualitative analysis) and mixed-method studies with a qualitative component

### Intervention/exposure

Telehealth interventions will be defined as any intervention provided at a distance using telecommunication networks as a medium to deliver rehabilitation care [12]. We will not distinguish between telehealth, telemedicine, telerehabilitation, telecare, or other similar terms. In this review telehealth will broadly include professional and patient interaction (online and offline) and delivery of pain management intervention at a distance (e.g., home exercises, pain education, self-management strategies, telephone counseling). It could comprise any combination of the following: one to one or group videoconferences, access to websites or apps, mobile and/or telephone. Studies that also present face-to-face or use written/paper-based material in combination with telehealth interventions will be included only when they report qualitative data specific to the telehealth component.

### Types of studies to be included

Qualitative studies (e.g., focus groups or individual interviews) and analysis methods (e.g., thematic analysis or phenomenological analysis) focused on exploring perceptions/experiences or attitudes of people with chronic musculoskeletal pain. Studies may include people who have engaged entirely or partially in telehealth interventions, e.g., people who started a telehealth intervention program and completed it; people who started a telehealth intervention and interrupted it. Mixed-method studies with a qualitative component will be included; only the qualitative data will be used for this review.

### Data extraction (selection and coding)

Two investigators will perform the title and abstract screening, and disagreements will be resolved by consensus or by a third investigator. The full text of eligible records will be retrieved and assessed by two investigators. Disagreement between the reviewers regarding the full text will be resolved initially by discussion and, if necessary, arbitration by a third reviewer. In case of insufficient or unclear information in a potentially eligible article, the authors will be contacted by email and a timeframe of 3 weeks to reply will be considered before article exclusion. Data extraction will be conducted by reviewers with previous experience in systematic reviews and qualitative research methodology.

We will extract the following data from included articles: author, year, country, design, data collection method, participant characteristics, type and description of the intervention, and qualitative information (themes/sub-themes) regarding enablers and barriers disclosed in results, discussion (if applicable), or annex/appendix sections.

### Critical appraisal of included studies

The critical appraisal skills program (CASP) guidelines will be used to appraise the methodological quality of the included studies (Table 2) [27]. Two investigators will assess the quality of the studies individually. Discrepancies between will be resolved by discussion. A third investigator will be consulted for arbitration if necessary.

**Table 2:** Criteria and coding used during Risk of Bias assessment following CASP checklist [27]

	Design	Criteria to assess risk of bias	Coding
Section A			
Are the results of the study valid?	1. Aims of the research	Clarity on every statement of which the research project was based on	Yes—it is clear what was the aim of the study and relevance Cannot tell—insufficient data No—lack of data on the aims of the study, importance and/or relevance on the studied topic
	2. Appropriate methodology	Adopted methodology to Conduct the study was well chosen according to the research question	Yes—research seeks to interpret the actions and/or subjective experiences of participants; qualitative Methodology is the central used to address research aims. Cannot tell—insufficient data No—inadequate interpretation on the actions and/or subjective experiences of participants; qualitative is not the right methodology to address research goal
Is it worth continuing?	3. Appropriate design	Selection of proper study design	Yes—researcher justifies and reveals the reason behind the choice of determined research design Cannot tell—insufficient data No—no information upon research design decision-making available
	4. Appropriate recruitment strategy	Description on the selection of patients’ procedure and explanation on eligibility criteria	Yes—clear explanation on how patients were selected and why this procedure was the most suitable to provide answers sought by the research; justification in case exclusion criteria Cannot tell—insufficient data No—lack of information upon eligibility criteria; unclear information regarding selection procedure
	5. Appropriate data collection	Data collection was done adequately to address the research issue	Yes—clear justification on data collection (e.g., focal group, semi-structured interview); clear explanation on methods used during interviews; documentation in case of changes in methods along the study (if yes: how and why); clear form of data (e.g., tape, video, notes); discussion upon saturation of data Cannot tell—insufficient data No—gaps or missing information on regarding setting, script, interview guide, implemented methods, form of data, sample size characteristics
	6. Consideration of relationship between researcher and participants	Consideration on influences faced along the development of the research and its potential consequences	Yes—critical examination on potential bias during formulation of research question, data collection, recruitment procedure and setting; clear explanation on how was the response to certain events during the study and implications for research design Cannot tell—insufficient data No—lack of discussion upon potential limitations and bias present in the study
Section B			
What are the results?	7. Ethical issues	Adherence to ethical standards	Yes—research successfully explained details for included patients; data upon informed consent, confidentiality or management of information during and after the study; consultation to ethical committee Cannot tell—insufficient data No—lack or poor information regarding actions taken according to ethical standards (informed consent, explanation on research aims to patients)
	8. Rigorous data analysis	Data analysis reporting is complete and detailed	Yes—clear in-depth description of analysis process, with explanation on how categories/themes were created from data analysis; sufficient data to support findings; accountability of contradictory data; critical examination on potential bias and influence during analysis and selection of data for presentation Cannot tell—insufficient data No—presented data is not supported by relevant information on how it was analyzed; gaps regarding development of themes/subthemes or/and missing explanation on the reason behind selection of certain data and potential bias
	9. Statement of findings	Clarity and adequate explanation on the findings	Yes—findings of the studies are explicit an there is proper discussion using evidence both for and against studies argument; findings are analyzed and checked regarding credibility; findings suits research main question Cannot tell—insufficient data No—findings are poorly explicit and there is a lack of discussion using evidence both agreeing and disagreeing with the findings; no credibility regarding findings was clearly stated; findings do not answer main research question
Section C			
Will the results help locally?	10. Relevance	Contribution research findings brings to communities	Yes—discussion is made upon impact of research on existing knowledge, policies, practices or relevant literature; study discuss new areas for future research are; researches discuss how the findings could be transferred to different populations or suggest different approaches for similar investigations Cannot tell—insufficient data No—no information regarding impact of research on existing literature, policies, and practices; study does not present any suggestion for future research directions; research does not discuss about how to transfer findings to different population

### Strategy for data synthesis

We will use a 3-step thematic synthesis method for data synthesis guided by an inductive approach [28]. The results section of the primary studies (verbatim) will be imported to NVivo or Microsoft Excel sheet. First, line-by-line coding of the results and discussion (if applicable) of the included articles will be performed. The discussion section will be coded only to provide additional contextual information if required. Subsequently, “descriptive themes” will be created based on the analysis of the results of the included studies. The last stage will involve generating “analytical themes” by studying each category and merging them in case of similarities, leading to the creation of major themes relevant to the key aim of this meta-synthesis [28]. Once the coding is complete, the research team will discuss the synthesis of findings and examine the derived major themes, aiming for consensus regarding the final themes.

The level of confidence for main findings from the meta-synthesis will be assessed using the confidence in the evidence from reviews of qualitative research (GRADE-CERQual) approach [29]. This method aims to assess the extent to which confidence can be placed in findings from qualitative synthesis. GRADE-CERQual approach is based on an assessment of the individual findings in terms of 4 components:

1. Methodological limitations of included studies—the extent to which there are concerns regarding the design of the primary studies that contributed to a review finding [30].
2. Coherence of review findings—assessment of how clear, well supported, and compelling is the communication between data from primary studies and a review finding addressing those data [31].
3. Adequacy of data contributing to a review finding—determination on how rich is the data supporting a review finding [32].
4. Relevance of included studies to the review question—to what extent the evidence from the primary studies support the review findings and its application to the context specified in the review question (focusing on perspective or population, phenomenon of interest, setting) [33].

This assessment will lead to judgment of the level of confidence in the evidence supporting each individual review finding [34]. The grading system is described as follows:

- *High confidence*. It is highly likely that the review finding is a reasonable representation of the phenomenon of interest.
- *Moderate confidence*. It is likely that the review finding is a reasonable representation of the phenomenon of interest.
- *Low confidence*. It is possible that the review finding is a reasonable representation of the phenomenon of interest.
- *Very low confidence*. It is not clear whether the review finding is a reasonable representation of the phenomenon of interest.

Each one of the 4 components will be categorized as follows: “no or very minor concerns,” “minor concerns,” “moderate concerns,” or “serious concerns”. All findings will start as high confidence and will be downgraded by one level if presenting “minor” and “moderate concerns;” evidence presenting “serious concerns” will be downgraded by two levels.

## Discussion

Achieving patient engagement in telehealth interventions is essential for positive outcomes but can be extremely challenging due to various barriers (e.g., cultural, social, economic). Despite existing evidence identifying telehealth interventions as a promising means of improving health dissemination to underserved populations, little is known about how best to deliver care to patients with chronic musculoskeletal pain remotely or using web-based sources. A qualitative evidence synthesis approach is best suited to address this research question. Systematic review of qualitative evidence allows an in-depth understanding of gaps from the point of view of the end users for whom the treatment is designed.

Using qualitative evidence synthesis brings relevant information on people’s perceptions, experiences, and opinions regarding interventions, health care services, policies, and processes [29]. When complemented by evidence related to effectiveness and costs, this information is better suited to informing decision-making and policy development, leading to improvements in implementation, practice, and health [29, 35].

This review will systematically identify, synthesize, and report the most common enablers and barriers for people with musculoskeletal pain to engage in telehealth interventions. The review will provide clear information required for designing and improving health services based on telehealth interventions and technology development.

## Data Availability

Not applicable

## Appendix

MEDLINE electronic search strategy

1 pain.mp.

2 “chronic pain”.mp. or exp Chronic Pain/

3 “persistent pain”.mp.

4 1 or 2 or 3

5 qualitative.mp. or exp Qualitative Research/

6 “focus group”.mp. or exp Focus Groups/

7 “grounded theory”.mp. or exp Grounded Theory/

8 phenomenology.mp.

9 “mixed method”.mp.

10 5 or 6 or 7 or 8 or 9

11 “self management”.mp. or exp Self Care/

12 pain management.mp. or exp Pain Management/

13 telemedicine.mp. or exp Telemedicine

14 tele health.tw. or tele*health.tw.

15 tele care.tw. or tele*care.tw.

16 e*health.tw. or ehealth.tw.

17 home*based.tw.

18 mobile health.tw. or mhealth.tw. or m health.tw. or m*health.tw.

19 telephone.tw. or exp Telephone/

20 smart phone.tw. or smart*phone.tw. or mobile phone.tw.

21 apps.tw.

22 exp Mobile Applications/

23 text messaging.tw. or exp Text Messaging/

24 exp Internet/ or Internet.tw.

25 internet*based.tw.

26 online.tw.

27 web based.tw. or webbased.tw.

28 computer based.tw.

29 videoconferencing.tw. or exp Videoconferencing/

30 tablet device.tw.

31 iPad.tw.

32 iPhone.tw.

33 distance.tw.

34 remotely delivered.tw.

35 11 or 12 or 13 or 14 or 15 or 16 or 17 or 18 or 19 or 20 or 21 or 22 or 23 or 24 or 25 or 26 or 27 or 28 or 29 or 30 or 31 or 32 or 33 or 34

36 4 and 10 and 35

Limit humans
